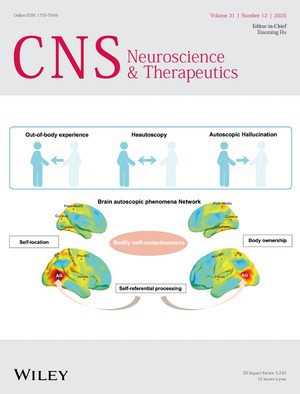# Front Cover

**DOI:** 10.1002/cns.70717

**Published:** 2025-12-22

## Abstract

Cover image: The cover image is based on the article *Brain Network Signature of Autoscopic Phenomena in Humans* by Siyi Wang et al., https://doi.org/10.1111/cns.70635.